# Supplementary material for: A shared neural code for the physics of actions and object events
Source: Nat Commun. 2023 Jun 7;14:3316. doi: 10.1038/s41467-023-39062-8 (PMC10247699; doi:10.1038/s41467-023-39062-8)
Supplement: Supplementary file 2 — Reporting Summary [file 41467_2023_39062_MOESM2_ESM.pdf]

nature portfolio

Corresponding author(s): Seda Karakose-Akbiyik

Last updated by author(s): Feb 28, 2023

Reporting Summary

Nature Portfolio wishes to improve the reproducibility of the work that we publish. This form provides structure for consistency and transparency in reporting. For further information on Nature Portfolio policies, see our Editorial Policies and the Editorial Policy Checklist.

Statistics

For all statistical analyses, confirm that the following items are present in the figure legend, table legend, main text, or Methods section.

n/a	Confirmed	
☐	☒	The exact sample size (n) for each experimental group/condition, given as a discrete number and unit of measurement
☐	☒	A statement on whether measurements were taken from distinct samples or whether the same sample was measured repeatedly
☐	☒	The statistical test(s) used AND whether they are one- or two-sided<br>*Only common tests should be described solely by name; describe more complex techniques in the Methods section.*
☐	☒	A description of all covariates tested
☐	☒	A description of any assumptions or corrections, such as tests of normality and adjustment for multiple comparisons
☐	☒	A full description of the statistical parameters including central tendency (e.g. means) or other basic estimates (e.g. regression coefficient) AND variation (e.g. standard deviation) or associated estimates of uncertainty (e.g. confidence intervals)
☐	☒	For null hypothesis testing, the test statistic (e.g. F, t, r) with confidence intervals, effect sizes, degrees of freedom and P value noted<br>*Give P values as exact values whenever suitable.*
☒	☐	For Bayesian analysis, information on the choice of priors and Markov chain Monte Carlo settings
☒	☐	For hierarchical and complex designs, identification of the appropriate level for tests and full reporting of outcomes
☐	☒	Estimates of effect sizes (e.g. Cohen's d, Pearson's r), indicating how they were calculated

Our web collection on statistics for biologists contains articles on many of the points above.

Software and code

Policy information about availability of computer code

Data collection	Stimulus presentation, response collection, and synchronization with the scanner were controlled with the MATLAB Psychtoolbox-3 for Windows.
Data analysis	BrainVoyager QX 2.8, Matlab 2018b, NeuroElf v1.0, CoSMoMVPA 1.1.0 (Oosterhof et al., 2016)

For manuscripts utilizing custom algorithms or software that are central to the research but not yet described in published literature, software must be made available to editors and reviewers. We strongly encourage code deposition in a community repository (e.g. GitHub). See the Nature Portfolio guidelines for submitting code & software for further information.

Data

Policy information about availability of data

All manuscripts must include a data availability statement. This statement should provide the following information, where applicable:

- Accession codes, unique identifiers, or web links for publicly available datasets
- A description of any restrictions on data availability
- For clinical datasets or third party data, please ensure that the statement adheres to our policy

Preprocessed neuroimaging data, design matrices, sample stimuli, and the Source data underlying Figs. 2-4 and Supplementary Figs. 3c, 6e, 6f are deposited at the Open Science Framework (https://osf.io/h4mtp/). Raw functional imaging data and the full set of stimuli are available from the corresponding author upon request.

Human research participants

Policy information about studies involving human research participants and Sex and Gender in Research.

Reporting on sex and gender	We collected data from 15 male and 10 female participants. The findings do not apply to only one sex or gender, and sex and gender were not considered in the study design. Sex and/or gender was determined based on self-reporting. We did not do any analyses based on gender or sex since our stimuli, experimental design, and/or questions did not have any relevance to sex and/or gender based differences.
Population characteristics	Twenty-five right-handed native Italian speakers participated in the experiment (Mage = 24.52, SDage = 4.80). All participants had normal or corrected-to-normal vision and no history of neurological or psychiatric disease.
Recruitment	Recruitment was based on the following inclusion criteria: right handers, Native Italian speakers, normal or corrected-to-normal vision, and no history of neurological or psychiatric disease. The subjects provided informed consent prior to the experiment. We recruited participants through a community announcement without any specific selection criteria besides the ones described above. In this case, it is unlikely that there was any intentional self-selection bias as participants were not specifically targeted or recruited based on any specific characteristics.
Ethics oversight	The participants provided informed consent before participation and all procedures were approved by the Ethics Committee for research involving human participants at the University of Trento, Italy.

Note that full information on the approval of the study protocol must also be provided in the manuscript.

Field-specific reporting

Please select the one below that is the best fit for your research. If you are not sure, read the appropriate sections before making your selection.

☒ Life sciences ☐ Behavioural & social sciences ☐ Ecological, evolutionary & environmental sciences

For a reference copy of the document with all sections, see nature.com/documents/nr-reporting-summary-flat.pdf

Life sciences study design

All studies must disclose on these points even when the disclosure is negative.

Sample size	No statistical test was used to predetermine sample size. We collected data from N = 25 participants who attended both the video and sentence sessions. Our previous work on human action observation showed that depending on region of interest, actions presented in videos can be decoded with sample sizes between N = 5 (left LOTC; with d=1.94, alpha = 0.05, power=0.95) and N = 14 (left PMC; with d=0.95, alpha = 0.05, power=0.95, see Wurm et al., Journal of Neuroscience, 2017). Another work from our lab showed cross-decoding of actions across verbal and visual stimuli with N = 22 participants (Wurm & Caramazza, Nature Communications, 2019). Given these considerations, we think that our sample size of N = 25 gives us sufficient power to identify where in the brain information about events are encoded and are in the range of sample sizes conventionally used in the field.
Data exclusions	Behavioral accuracies 2 SD below the average were used as a pre-established exclusion criterion. Based on this criterion, no participant was excluded from the sample.
Replication	We searched for a shared neural code for actions and object events by using both visual and verbal stimuli. Even though these two studies provided complementary evidence for one another, no further replication was attempted. Furthermore, our results from human action decoding gave us a chance to evaluate our novel approach against previous work and revealed effects that are consistent with what we already know about the human brain. For instance, decoding of actions presented in videos and cross-modal decoding in previous results were reliably reproduced (see Wurm et al., Journal of Neuroscience, 2017; Wurm & Caramazza, Nature Communnications, 2019).
Randomization	Session order was balanced across participants (odd IDs = video first, even IDs = sentence first). Conditions were first order counterbalanced within experimental runs for each participant.
Blinding	Functional data were collected by M.W., who is an author on the paper and was aware of the different experimental conditions. Data analyses were not performed blind to the conditions of the study as specification of different experimental sessions and motion events were crucial for our neuroimaging analyses, and their interpretation, particularly for decoding.

Reporting for specific materials, systems and methods

We require information from authors about some types of materials, experimental systems and methods used in many studies. Here, indicate whether each material, system or method listed is relevant to your study. If you are not sure if a list item applies to your research, read the appropriate section before selecting a response.

Materials & experimental systems

n/a	Involved in the study
☒	☐ Antibodies
☒	☐ Eukaryotic cell lines
☒	☐ Palaeontology and archaeology
☒	☐ Animals and other organisms
☒	☐ Clinical data
☒	☐ Dual use research of concern

Methods

n/a	Involved in the study
☒	☐ ChIP-seq
☒	☐ Flow cytometry
☐	☒ MRI-based neuroimaging

Magnetic resonance imaging

Experimental design

Design type: mixed event-related design

Design specifications: The video and sentence experiments consisted of four and five functional scans, respectively. Each functional scan started with a 10 s fixation period and ended with a 16 s fixation period. Four blocks were presented per run, separated by 10 s fixation periods. Twenty-eight trials were shown per block. In the video session, for each of the 6 unique events, there were 64 trials in total (4 trials per block X 4 blocks per run X 4 runs per session). For sentences, for each of the 6 unique events, there were 80 trials in total (4 trials per block X 4 blocks per run X 5 runs per session. In every stimulus trial, videos or sentences (2 s) were followed by a 1 s fixation period.

Behavioral performance measures: accuracy and false alarm rate on catch trial detection; button press using right index finger

Acquisition

Imaging type(s): functional, structural

Field strength: 3T

Sequence & imaging parameters: Neuroimaging data were acquired using a 3T Siemens Prisma fMRI Scanner with a 32-channel phased-array head coil. T1-weighted structural images were obtained using a 3D MPRAGE sequence (176 sagittal slices; repetition time (TR) = 2530 ms; inversion time = 1020 ms; flip angle = 7 degrees; field of view (FoV) = 256 x 256 mm; 1x1x1 mm voxel resolution). Blood oxygenation level-dependent (BOLD) contrast functional images were obtained using a T2*-weighted gradient echo-planar imaging (EPI) sequence (TR = 1500 ms; echo time (TE) = 28 ms; inter slice time = 33 ms; flip angle = 70 degrees; FoV = 200 mm x 200 mm; matrix size = 66 x 66; 3x3x3 mm voxel resolution; 45 slices with 3 mm thickness and 0 mm gap).

Area of acquisition: whole brain

Diffusion MRI ☐ Used ☒ Not used

Preprocessing

Preprocessing software: BrainVoyager QX 2.8, NeuroElf v1.0

Normalization: Functional and structural data were normalized using trilinear interpolation.

Normalization template: Talairach

Noise and artifact removal: Distortions in geometry and intensity in the echo-planar images were corrected on the basis of the PSF data acquired before each EPI scan. We preprocessed and analyzed functional and anatomical data using BrainVoyager QX 2.8 (BrainInnovation) in combination with the BVQXTools, NeuroElf Toolboxes and MATLAB (MathWorks) functions. The first four volumes of functional runs were removed to prevent T1 saturation. Preprocessing of functional data included slice time correction, three-dimensional motion correction (trilinear interpolation, the first volume of the first run of each participant was used as reference), linear trend removal, high-pass filtering (cutoff frequency of three cycles), and spatial smoothing (Gaussian kernel of 8mm FWHM for univariate analyses and 3 mm FWHM for MVPA). Functional images were registered to high-resolution anatomical images (six parameters), and anatomical and functional data were normalized to Talairach space.

Volume censoring: No volume censoring was performed.

Statistical modeling & inference

Model type and settings: Single-subject level: MVPA (support vector machine (libsvm), C-SVC, linear), 12 mm spherical searchlight), RSA-GLM
Between-subject level: Whole-brain analyses - random effects t-test. ROI analyses - linear mixed effects models nested within subjects, estimated marginal means for post-hoc contrasts. All p-values were adjusted for multiple testing by using the FDR method.

Effect(s) tested	One-tailed t tests to investigate above chance classification of events for human actions and object events, and by generalizing across animacy (i.e., train with actions, test with object events, and vice versa), and modality (i.e., train with videos, test with sentences) in the whole brain. Two-tailed estimated marginal means comparisons to compare decoding accuracies for within-actions and within-object-events decoding in the whole brain. Linear mixed effects models for ROI and event type interactions, post-hoc contrasts to investigate the conditions driving the interactions All p-values were adjusted for multiple testing by using the FDR method.

Specify type of analysis: ☐ Whole brain ☐ ROI-based ☒ Both

Anatomical location(s)	ROIs were independently determined based on a meta-analysis of action observation (Caspers et al., 2011). Five ROIs were picked in each hemisphere: lateral occipitotemporal cortex, ventral premotor cortex, anterior inferior parietal lobe, superior temporal sulcus, superior parietal lobe. For the SPL ROI, Caspers et al. (2010) provides multiple coordinates, and since we did not have an a priori reason for selecting one over the other, we selected the centroid of the Brodmann area 7 for SPL to maintain consistency across the two hemispheres. The following Talairach coordinates were used: left LOTC [-45 -71 6], left IPL [-58 -23 34], left PMv [-48 8 29], left pSTS [−52 −49 11], left SPL [-18 -57 50]; right LOTC [52 -63 5], right IPL [44 -31 41], right PMv [50 12 27], right pSTS [54 −40 8], right SPL [24 -56 54].

Statistic type for inference (See Eklund et al. 2016)	Cluster-based, 10000 Monte Carlo simulations
Correction	Whole-brain: Monte Carlo Cluster correction (as implemented in CoSMoMVPA 1.1.0; Oosterhof et al., 2016); ROI-based: FDR correction

Models & analysis

n/a	Involved in the study
☒	☐ Functional and/or effective connectivity
☒	☐ Graph analysis
☒	☐ Multivariate modeling or predictive analysis
